# Analysis of PEM Water Electrolyzer Failure Due to Induced Hydrogen Crossover in Catalyst-Coated PFSA Membranes

**DOI:** 10.3390/membranes13030348

**Published:** 2023-03-17

**Authors:** Eveline Kuhnert, Mathias Heidinger, Daniel Sandu, Viktor Hacker, Merit Bodner

**Affiliations:** 1 Institute of Chemical Engineering and Environmental Technology, Graz University of Technology, Inffeldgasse 25/C, 8010 Graz, Austria; 2 AiDEXA GmbH, Bergmanngasse 45/10, 8010 Graz, Austria

**Keywords:** polymer electrolyte membrane water electrolysis (PEMWE), membrane degradation, fluoride emission rate (FER), hydrogen crossover, accelerated stress test (AST)

## Abstract

Polymer electrolyte membrane water electrolysis (PEMWE) is a leading candidate for the development of a sustainable hydrogen infrastructure. The heart of a PEMWE cell is represented by the membrane electrode assembly (MEA), which consists of a polymer electrolyte membrane (PEM) with catalyst layers (CLs), flow fields, and bipolar plates (BPPs). The weakest component of the system is the PEM, as it is prone to chemical and mechanical degradation. Membrane chemical degradation is associated with the formation of hydrogen peroxide due to the crossover of product gases (H_2_ and O_2_). In this paper, membrane failure due to H_2_ crossover was addressed in a membrane-focused accelerated stress test (AST). Asymmetric H_2_O and gas supply were applied to a test cell in OCV mode at two temperatures (60 °C and 80 °C). Electrochemical characterization at the beginning and at the end of testing revealed a 1.6-fold higher increase in the high-frequency resistance (HFR) at 80 °C. The hydrogen crossover was measured with a micro-GC, and the fluoride emission rate (FER) was monitored during the ASTs. A direct correlation between the FER and H_2_ crossover was identified, and accelerated membrane degradation at higher temperatures was detected.

## 1. Introduction

Green hydrogen has great potential as a key element in energy transition, as it is relatively easy to store, can be produced in a variety of ways, and is CO_2_-neutral when produced by electrolysis with green electricity [1]. Over the past years, electrolyzers have attracted more and more interest due to the progress in hydrogen technologies such as fuel cells for mobile and stationary applications [2]. Compared to other technologies used for hydrogen production, polymer electrolyte membrane water electrolyzers (PEMWEs) are reliable for a wide range of applications and can be implemented in renewable energy systems [3,4,5]. They are highly efficient, and state-of-the art PEMWEs can operate at above 3 A cm^–2^ and have quoted lifetimes of up to 100,000 h [6].

The proton-conducting polymer electrolyte membrane (PEM) is the major component of a PEMWE and is used as a product gas (O_2_ and H_2_) separator, electrolyte, and support for the cathode- and anode catalyst layers (CLs) [7]. Therefore, membranes used in PEMWEs must be dimensionally stable; proton conductive; mechanically, chemically, and thermally stable; and have sufficient gas-crossover resistance [7,8,9]. In PEMWE operation, the supplied water is decomposed into protons and oxygen in the anode’s Ir-based CL. The Ir-based catalysts are required on the anode of the electrolyzer due to the harsh environment of the oxygen evolution reaction (OER). The hydrogen protons pass through the PEM to the Pt/C cathode CL, where they combine with electrons to build gaseous H_2_. The cathodic half-cell reaction is also known as a hydrogen evolution reaction (HER).

**Figure 1:**
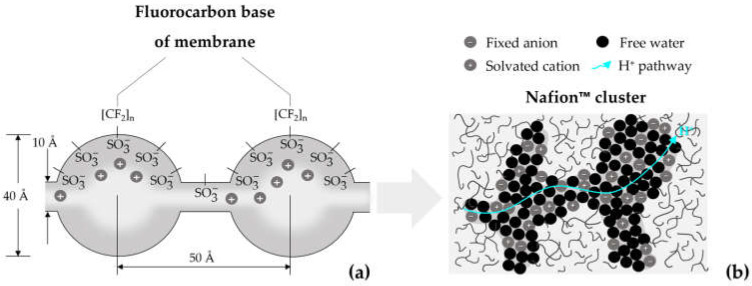
Structure model of Nafion™ in PEM water electrolysis (PEMWE): cluster-channel (**a**) and H^+^ pathway through a Nafion™ cluster surrounded by hydrophobic polymer chains (**b**).

Generally, PFSA-based materials such as Nafion™ membranes of different thicknesses are used in PEMWEs. Nafion™ consists of a hydrophobic Teflon-like backbone and hydrophilic sulfonic-acid-terminated side chains that provide protonic conductivity [10,11]. The structure model of a Nafion™ membrane is represented in Figure 1. According to the theory of ion-clustering by Gierke [12], the sulfonic-acid-side chains in Nafion™ are organized in clusters of ~40 Å (Figure 1a). The H^+^ protons are transferred through narrow, water filled channels by means of the sulfonic-acid-terminated side chains [13,14]. Hydrophobic polymer regions enhance the H^+^ mobility in the membrane cluster-channels (Figure 1b) [14].

Membrane humidification is crucial for the protonic conductivity (~0.1 S·cm^−1^) of Nafion™ membranes. The targeted high operating temperatures in PEMWE influence the water uptake and add stress on the membrane. At high temperatures, the hydrogen crossover rate also increases due to the enhanced hydrogen permeability of the PEM [15,16]. Especially in idle operation, hydrogen crossover is increased due to asymmetric H_2_O and gas pressure ratios. As a result, membrane degradation and thus thinning and pinhole formation can occur due to the formation of radicals and, potentially, hydrogen peroxide at the CLs [15,17]. Different reactions are involved in the formation of harmful species from H_2_ and O_2_ in PEM technology. Initially, hydrogen radicals are produced from H_2_ in the presence of Pt on the cathode CL. The H• radicals can diffuse through the PFSA membrane and react with O_2_ on the anode side of the electrolyzer to HO_2_• (see reactions I and II). HO_2_• can then either directly attack the ionomer or recombine with H• to form hydrogen peroxide (reaction III). Radical-forming metal cations such as Fe^2+^ or Cu^2+^ found in PEMWE membrane electrode assemblies (MEAs) result in the formation of •OH radicals (reaction IV). In a last step, free hydrogen peroxide radicals are formed that attack the PEM and accelerate membrane degradation (reaction V) [18].

H_2_ → 2H• (I)

H• + O_2_ → HO_2_• (II)

HO_2_• + H• → H_2_O_2_ (III)

H_2_O_2_ + M^2+^ → M^3+^ + •OH + OH^–^ (IV)

•OH + H_2_O_2_ → H_2_O + HO_2_• (V)

Degradation effects in PEMs for water electrolysis have been studied by several researchers [15,17,19,20,21,22]. Fouda-Onana et al. [21] developed an accelerated stress test (AST) in which they cycled a PEMWE single cell between 0 and 1 A·cm^–2^. They measured the fluoride emission rate (FER) as an indicator of membrane degradation and found that the FER shows a maximum between 0.2 and 0.4 A·cm^−2^. A competitive reaction between radical (•OH) formation and two reactions that consume radicals was considered as the reason for the high FER in the low-current density region. The researchers further investigated the impact of temperature (60 °C and 80 °C) and current density (galvanostatic profile between 0 to 1 A·cm^−2^) on membrane degradation, and the FER showed a 5-fold increase at 80 °C [21]. Frensch et al. [17] investigated the impact of different operation modes on PEMWE degradation. The greatest influence on membrane degradation was observed at 90 °C, where the FER and membrane thinning increased significantly. The researchers concluded that the high temperature operation increased the hydrogen crossover and therefore was responsible for membrane degradation over time [17].

For PEMFCs, a technology facing related problems regarding degradation, harmonized test protocols for the evaluation of the performance and durability of MEA components exist [23,24]. The department of energy (DoE) suggested a MEA chemical stability AST at a steady-state open circuit voltage (OCV) and membrane mechanical cycle metrics for the acceleration of membrane failure [24]. 

Based on the understanding of membrane degradation in PEMWE, a novel stress test protocol was developed in the present work. Hydrogen crossover through the PFSA membrane was induced through asymmetric H_2_ (cathode) and O_2_/H_2_O (anode) gas and water supply to a PEM single-cell electrolyzer in idle conditions. Different to ASTs proposed in the literature, higher degradation can be achieved in a short period of time due to the increased crossover rates at steady-state OCV. Additionally, two different temperature setpoints (60 °C and 80 °C, respectively) were tested to evaluate the severity of the temperature in the proposed test protocol. A set of electrochemical measurements was employed at the begin of testing (BoT) and at the end of testing (EoT). Electrochemical impedance spectroscopy (EIS) was used to determine the high-frequency resistance (HFR) and low-frequency resistance (LFR) of the membranes, and polarization curves were recorded to measure the cell’s performance. The H_2_ in O_2_ content in the anode gas stream was analyzed with gas chromatography (GC) to obtain an indication of the hydrogen crossover rate. Furthermore, the anode-circulating water was sampled periodically during the ASTs to determine the FER. To our knowledge, no similar AST protocol has been reported in the literature for the acceleration of chemical membrane degradation.

## 2. Materials and Methods

### 2.1. Test Setup and Cell Assembly

**Figure 2:**
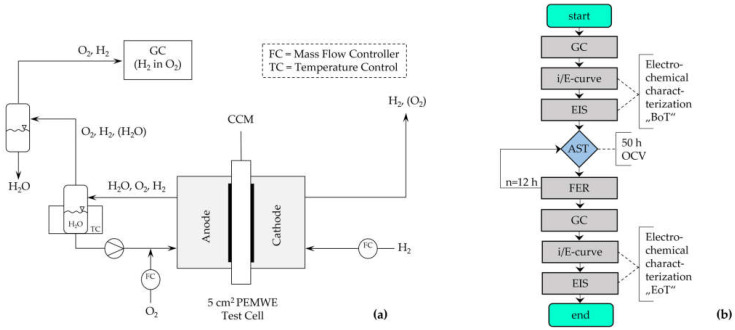
Schematic representation of the test setup (**a**) and measurement protocol (**b**). Abbreviations: AST = accelerated stress test; BoT = begin of testing; CCM = catalyst coated membrane; EIS = electrochemical impedance spectroscopy; EoT = end of testing; FER = fluoride emission rate; GC = gas chromatography; OCV = open circuit voltage.

Commercial Nafion™ 117-based CCMs (CCM-E05-N117, Quintech, Göppingen, Germany) with an active area of 5 cm^2^ were used in this study. The cathode CL loading of the CCMs was 1.0 mg·cm^−2^ Pt/C, and the anode CL loading was 2.0 mg·cm^−2^ Ir. Gas diffusion media Ti-felt PTLs (Ti grade 1, ~350 µm thickness, Bekaert, Belgium) were used on both sides of the CCM. All experiments were carried out in an in-house-built test cell with single serpentine flow fields, polyolefine sealings (Ice Cube, Freudenberg, Germany, Weinheim), and stainless-steel endplates. The test cell was heated with an external heating bath (Julabo, Seelbach, Germany) and connected to a test station with gas (H_2_ and O_2_) and water supply (ultrapure H_2_O, resistivity > 18 MΩ·cm). The gas flow rates were controlled with mass flow controllers for H_2_ and O_2_ (Bronkhorst, Veenendal, Netherlands), and the water flow rate was set with a micro annular gear pump (Faulhaber, Schönaich, Germany). A schematic of the test setup is presented in Figure 2a. 

H_2_ (purity grade 5.0 [≥ 99.999%] 350 mL·min^−1^) was supplied to the cathode side, and O_2_ (purity of grade 4.5 [≥ 99.995%] 20 mL·min^−1^) was supplied with intermittent water injection to the anode side of the test cell. The hydrogen crossover was determined by measuring the H_2_ in O_2_ content of the dried anode outlet stream with a gas chromatograph (micro-GC fusion gas analyzer, Inficon, Switzerland). 

### 2.2. Membrane Accelerated Stress Test

The membrane-focused AST consisted of 50 h in OCV mode at 60 °C and 80 °C, respectively. During this idle period, high H_2_ gas flow (350 mL·min^−1^) was supplied to the cathode-side of the electrolyzer, and O_2_ (20 mL·min^−1^) was supplied with intermittent H_2_O injections (5 mL·min^−1^) to the anode side of the test cell. The effluent water was collected periodically every 12 h during the measurements. The anode-circulating water flow was used to determine the FER from the membrane during the AST period. From the dried anode exhaust gas stream, the hydrogen crossover (H_2_ in O_2_) was measured by a micro-GC. The sequence of the AST is displayed in Figure 2b.

### 2.3. Performance Characterization

After cell assembly with fresh MEAs, a conditioning step was applied. To ensure full humidification of the membrane, the test cells were conditioned by recirculating the anode feed water at the operating temperature (60 °C and 80 °C) for six hours. Afterwards, galvanostatic hold steps were applied at 0.2 A·cm^−2^ and 1.0 A·cm^−2^ for 30 min, respectively. After the initial conditioning step, polarization (i/E) curves and EIS measurements were conducted to record the cell performance at BoT. The i/E-curves were measured between 0.01 and 2 A·cm^−2^ as described in the EU harmonized polarization curve test protocols for low-temperature water electrolysis [25]. EIS was measured at two current densities (0.2 and 1 A·cm^−2^) with a frequency swept from 100,000 to 0.1 Hz and an amplitude 5–10% of the applied current. During the polarization curves and EIS measurements, only H_2_O (ultrapure H_2_O, resistivity >18 MΩ·cm, 5 mL·min^−1^) was supplied to the anode compartment of the cell.

### 2.4. Characterization Techniques

#### 2.4.1. Hydrogen Crossover

The hydrogen crossover (H_2_ in O_2_) was determined at three different current densities (0.2, 0.5 and 1 A·cm^−2^) during electrolyzer operation. The dwell time for each current step was based on previous studies, in which each current density was held until a constant value for the hydrogen content was reached [15,26]. The dried anode gas stream was analyzed via GC at ambient pressure. Multiple-level calibration of the micro-GC was carried out by running sample calibration standards of different concentration levels (H_2_ and O_2_). The measurement procedure was proceeded twice for each current density step.

#### 2.4.2. Fluoride Emission Rate

The fluoride emission from the membrane was monitored with a photometric method, as recently described by our group [27]. Therefore, 100 µL of Zr(IV)-SPADNS2 (Merck, Darmstadt, Germany) was added to 900 µL of each sample. An UV-VIS spectrometer (AiDEXA GmbH, Graz, Austria) [28] was used to record absorption spectra. The fluoride content (${µg}_{F^{-}}$h^−1^cm^−2^) was determined from the apparent molar absorptivity of the quenched Zr(IV)-SPADNS2 complex by means of a standard calibration curve. The standard calibration curve was prepared from analysis grade NaF (Merck, Darmstadt, Germany) and ultrapure H_2_O (>18 MΩ·cm).

#### 2.4.3. Scanning Electron Microscopy

Scanning Electrode Microscopy (SEM) analysis was carried out on two aged and one fresh CCM with a FEI-XL20 (Philips, Amsterdam, the Netherlands). Therefore, the CCMs were cut into squares of ~0.5 × 0.5 cm^2^ and placed on stainless-steel stubs with conductive double-sided adhesive carbon tabs. Furthermore, cross-sectional images were recorded of the fresh and used CCMs by cutting the samples with a sharp scalpel and placing them in specific sample holders for vertical analysis. 

## 3. Results and Discussion

### 3.1. Performance Characterization

**Figure 3:**
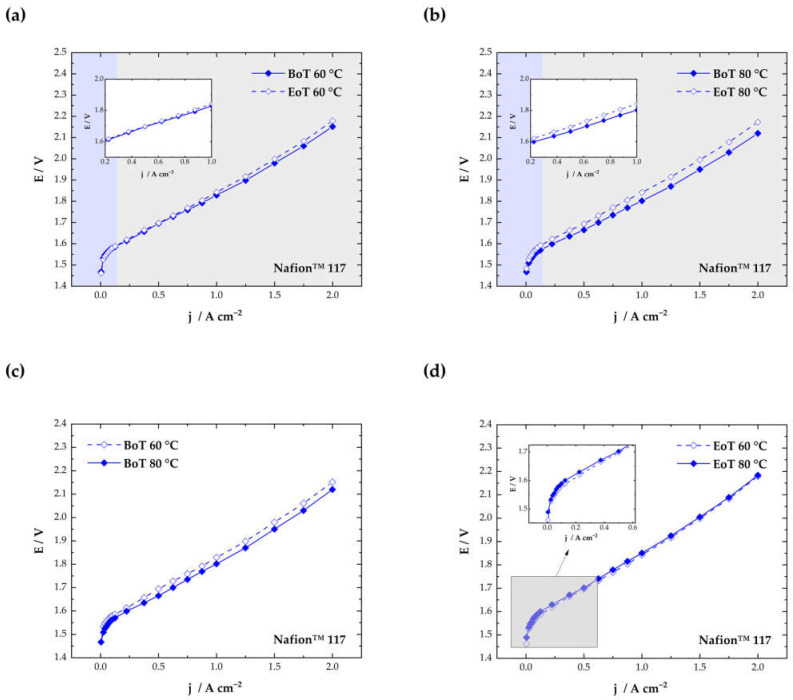
Polarization curves at begin of testing (BoT) and end of testing (EoT) for cells tested at 60 °C (**a**) and 80 °C (**b**) and performance comparison between 60 °C and 80 °C at BoT (**c**) and EoT (**d**).

The cells were electrochemically characterized with polarization curves and EIS measurements (0.2 and 1.0 A·cm^−2^) after the conditioning step at BoT and after the ASTs (EoT). In Figure 3, the polarization curves for the cell tested at 60 °C (a) and for the cell tested at 80 °C (b) are displayed. The main losses that dominate the shape of the polarization curves in PEMWE are kinetic, ohmic, and mass transport losses. Kinetic losses are mainly attributed to OER on the anode and result in an exponential increase in the cell voltage at low current densities (blue regions in Figure 3a−b).

The ohmic regions, marked grey in Figure 3a−b, can be seen in a linear increase in the cell voltage between approximately 0.5 and 2.0 A·cm^−2^. The ohmic region is dominated by the ion transfer resistance of the membrane. Compared to PEMFC, where thin membranes are used (<50 µm), thicker materials are necessary in electrolyzer operations (~100 µm) to ensure low gas crossovers and mechanical stability [29]. Therefore, ohmic losses are much more pronounced in PEMWE. From the polarization curves measured at 80 °C (Figure 3b), a clear increase in the kinetic and ohmic region can be observed after testing.

Generally, the performance of the cell at BoT is comparable to results from the literature (2.12 V at 2 A·cm^−2^ at 80 °C) [30]. At 80 °C, a better performance can be observed, compared to 60 °C (see Figure 3c). This is due to enhanced conductivity of the membrane at higher temperatures and better reaction kinetics for HER and OER [31].

**Figure 4:**
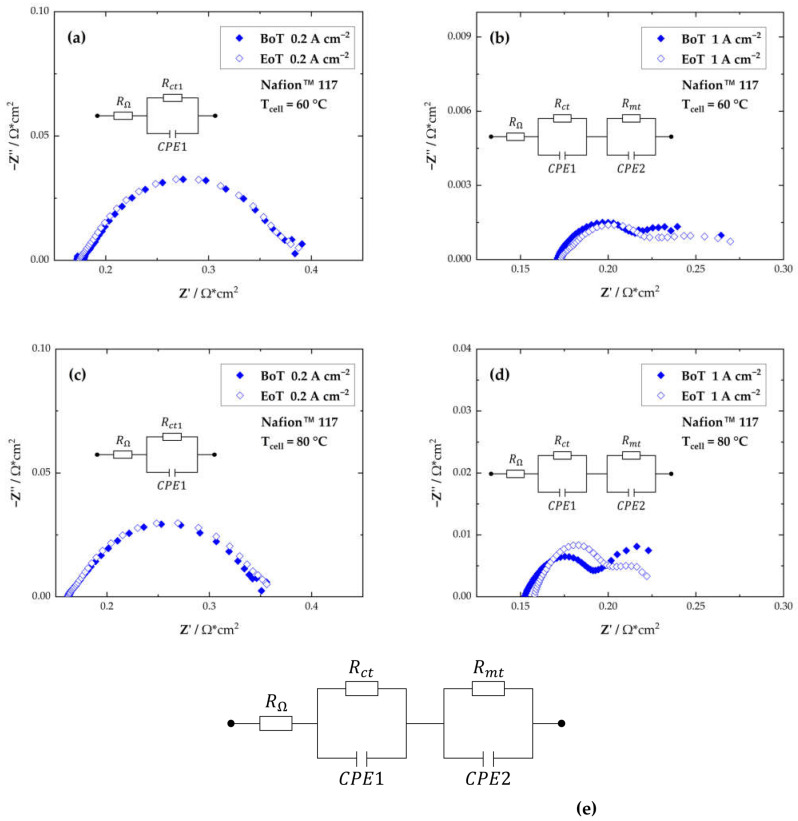
Nyquist plots recorded at 60 °C (**a**,**b**) and 80 °C (**c**,**d**) at two different current density setpoints; and the equivalent circuit model used for data evaluation (**e**).

A comparison of the cell performance at EoT in Figure 3d shows a more pronounced increase in the cell voltage in the kinetic region at 80 °C (highlighted inset in Figure 3d). This might indicate a change in the anode CL after the AST conducted at 80 °C. At higher current densities, mass transport losses become visible as a result of the limited diffusion of the reactants through the porous transport layers. This behavior is visible at higher currents in the Nyquist plots in an additional semicircle at low frequencies (Figure 4b,d).

The Nyquist plots measured at 60 °C and 80 °C and at two different current density setpoints are displayed in Figure 4. For further analysis of the EIS data, an equivalent circuit model was used (Figure 4e). The model consisted of a resistor (R_Ω_) representing the ohmic resistance and two resistors with constant phase elements in series (the charge transfer resistance, R_ct_ and the mass transport resistance, R_mt_). As expected, at the higher temperature of 80 °C, the overall resistance, and therefore also the R_ct_, are lower than at 60 °C due to improved reaction kinetics [32]. Compared to the R_ct_ dominating the EIS spectra recorded at 60 °C for BoT and EoT, at 80 °C the R_ct_ is the predominant factor after testing. This indicates the highest catalyst activity at BoT at 80 °C, but also the most detrimental impact of the changes of the catalyst layer activity after the AST was conducted at 80 °C. For a comparison reason, the slope of the polarization curve between 0.6 and 1.4 A·cm^−2^ was compared to the HFR from the EIS data recorded at 1.0 A·cm^−2^. Additionally, the LFR, representing the total resistance of the cell was determined from the Nyquist plots.

The ohmic resistances deducted from the slopes in the ohmic region of the polarization curves and the HFR and LFR at 1.0 A·cm^−2^ are listed in Table 1. The results from the EIS analysis imply a good agreement with the EIS data and the slope determined from the polarization curves, which indicates the reproducibility of the test rig, the PEM electrolyzer cell and the measurement system. At 80 °C the values from the slope of the i/E curve and the HFR determined from the Nyquist plots, representing the ohmic resistance of the cell, align perfectly. The slightly lower values at 60 °C from the i/E data might be explained by the non-isothermal behavior of the i/E-curves [33,34].

**Figure 5:**
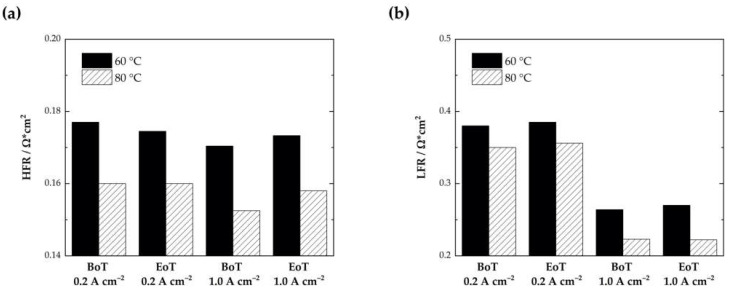
Comparison of the high-frequency resistance, HFR (**a**) and the low-frequency resistance, LFR (**b**) for the cells tested at 60 °C and 80 °C at the beginning of testing (BoT) and at the end of testing (EoT) and at two different current densities.

The HFR and LFR are additionally plotted in Figure 5 for the cells at 60 °C and 80 °C at BoT and at EoT. As expected, the LFR determined at approximately 1 HZ was lower at 80 °C (0.26 Ω·cm^2^ at 60 °C compared to 0.23 Ω·cm^2^ at 80 °C). No significant changes between the LFRs at BoT and EoT could be determined. The LFR increases at 60 °C and 80 ° by only ~1%. At 60 °C from 380.0 to 385.1 mΩ·cm^2^ at 0.2 A·cm^−2^ and from 264.0 to 270.1 mΩ·cm^2^ at 1.0 A·cm^−2^ and at 80 °C from 350.2 to 356.0 mΩ·cm^2^ at 0.2 A·cm^−2^ and from 223.3 to 222.2 mΩ·cm^2^ at 1.0 A·cm^−2^.

The cell tested at 80 °C experiences an increase in the HFR at both current densities (from 159.7 to 160.3 mΩ·cm^2^ at 0.2 A·cm^−2^ and from 152.5 to 158.0 mΩ·cm^2^ at 1.0 A·cm^−2^). The increased HFR after the AST matches the behavior visible in Figure 3b. The voltage increases from 1.80 to 1.84 V at 1 A·cm^−2^, which indicates a performance decay by ~2.2%. For the cell tested at 60 °C, no clear trend can be observed. At 0.2 A·cm^−2^, the HFR is reduced (from 177.5 to 174.5 mΩ·cm^2^), which could indicate an increased catalyst utilization according to the literature [35]. At 1.0 A·cm^−2^, only a slight increase in the HFR can be observed (from 170.4 mΩ·cm^2^ to 173.3 mΩ·cm^2^). Results from the polarization curves, however, indicate a transition point at approximately 0.5 A·cm^−2^ (displayed in the highlighted insets in Figure 3a). Until this point, the operational voltage seems to be slightly lower, which could indicate a better activation of the catalyst layer in the kinetic region, which is mainly dominated by the OER. From 0.5 A·cm^−2^ upwards, a slight voltage decay becomes visible (~0.8% from 1.83 to 1.84 V at 1 A·cm^−2^).

Compared to traditional PEMWE operation, where water is fed to the anode compartment of the electrolyzer cell to produce hydrogen and oxygen, the present work induces a significantly higher rate of membrane degradation. A reference protocol that consisted of a 1.3 V potentiostatic control during OCV was performed by Weiß et al. [4] on a 5 cm^2^ single cell PEMWE at ambient pressure and at 80 °C. In the first 200 cycles of their study (approximately 120 h of testing), the HFR even decreased at 1 A·cm from ~52 mΩ·cm^2^ to ~48 mΩ·cm^2^, whereas the AST protocol presented in this study resulted in an increase in the HFR from ~153 mΩ·cm^2^ to ~158 mΩ·cm^2^ at 80 °C after only 50 h of testing [4]. The overall lower membrane resistance in the reference test performed by Weiß et al. [4] can be explained by the use of a thinner membrane (Nafion™ 212, 50 µm).

### 3.2. Hydrogen Crossover

A constant O_2_ gas flow was reached after approximately 15 min for the lower current densities (0.2 and 0.5 A·cm^−2^) and after 10 min for the galvanostatic hold step at 1 A·cm^−2^. For the evaluation of the H_2_ concentration in the O_2_ stream, the hydrogen volume fraction in the dried anode outlet stream can be expressed by Equation (1). Here, $N_{H_{2}}^{GC}$ is the hydrogen crossover flux, and $\varphi_{H_{2}}^{GC}$ is the H_2_ volume fraction of the dried product stream ($N^{GC,out}$) [36].

(1) $$\varphi_{H_{2}}^{GC}=\frac{N_{H_{2}}^{GC}}{N^{GC,\;out}}$$

For the determination of the hydrogen content $\varphi_{H_{2}}^{cross}$ the evolved oxygen flux $N_{O_{2}}^{evo}$ is considered in Equation (2).

(2) $$\varphi_{H_{2}}^{cross}=\frac{N_{H_{2}}^{cross}}{N_{O_{2}}^{evo}+N_{H_{2}}^{cross}}$$

**Figure 6:**
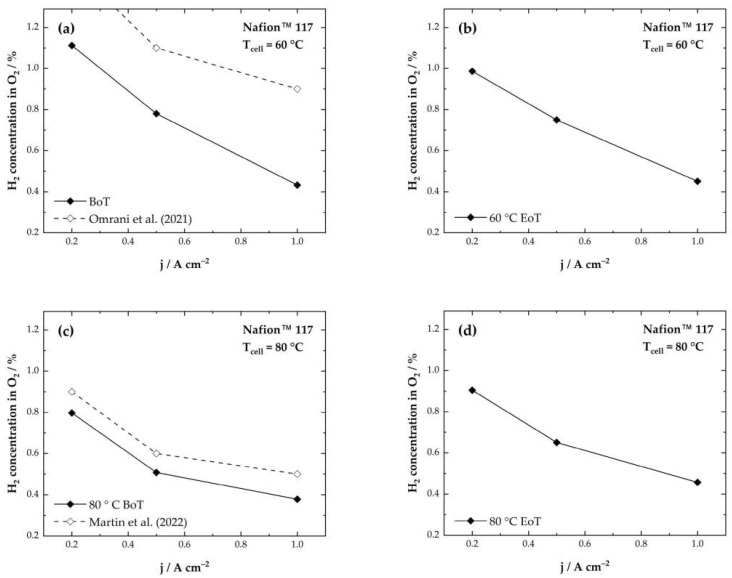
Hydrogen concentrations in oxygen for Nafion™ 117 CCMs in electrolyzer operation at 1 bar and 60 °C (**a**,**b**) and 80 °C (**c**,**d**) and comparison to those in the literature.

At very low current densities, only little oxygen is produced, which might be responsible for the sharp rise of the H_2_ in O_2_ concentration at 0.2 A·cm^−2^. Figure 6a,b shows the measured H_2_ content in the O_2_ stream for the cell tested at 60 °C. Interestingly, a significant decrease in the H_2_ concentration at 0.2 A·cm^−2^ from 1.11% to 0.9% can be observed after 50 h of testing. For the higher current densities, only a slight deviation is observed (see Table 2). 

Concerning hydrogen permeation, a transient overshoot of the hydrogen-in-oxygen concentration in the anode is predicted at lower current densities. This effect is based on the short-term mass storage capacity of dissolved supersaturated H_2_ in the membrane [37]. Therefore, changes in the membrane catalyst layer might be responsible for the lowered hydrogen crossover rate at 0.2 A·cm^−2^ at the end of testing. The values reported in this work for the cell tested at 60 °C are lower than the values reported in the literature (Figure 6a) [16]. This might be due to different membrane materials and thicknesses used in the experiments. The H_2_ crossover has also been shown to have a correlation with membrane temperature and humidification. 

At higher temperatures at 80 °C, an almost linear increase in the H_2_ concentration can be observed for all current densities after the AST (Figure 6c,d). As the humidification is constant for both temperature setpoints, and the membrane should be fully humidified due to the exposure to liquid water, the temperature will be the differentiating factor for gas crossover. Therefore, the dominating factor increasing the H_2_ crossover is the chemical degradation of the membrane. This also correlates with the increasing HFR after the experiments. 

Generally, the H_2_ concentrations displayed in Figure 6 show a maximum at 0.2 A·cm^−2^ for both cells. This behavior has been described in previous literature [15,26,38,39]. In Table 2, the initial H_2_ in O_2_ concentrations (BoT) and the H_2_ in O_2_ concentrations after the ASTs (EoT) are listed together with values reported in literature. A comparison with results from the literature shows that our results are in the range of values reported before for PEMWE. 

Omrani et al. [16] considered reported findings from literature on gas crossover in PEMFCs and developed a model that is applicable for PEM electrolyzers. The model includes several parameters like hydrogen pressure, hydrogen supersaturation, temperature, and compression [16]. At 60 °C and 1 bar their model revealed slightly higher values for the H_2_ concentration in the O_2_ stream than we have shown in this work (at 0.2 A·cm^−2^ ~1.5% H_2_ in O_2_ compared to 1.1% H_2_ in O_2_ in this work). 

Martin et al. [15] correlate hydrogen crossover with the supersaturation of hydrogen on the cathode. They analyzed the hydrogen crossover in PEMWE cells at ambient and elevated conditions. According to the researchers, mass transport limitations that depend on the applied current density are responsible for the supersaturation [15]. At 1 bar and 0.2 A·cm^−2^ they measured ~0.9% H_2_ concentration in the O_2_ stream compared to 0.8% H_2_ in O_2_ concentration in this work.

**Table 2:** H_2_ concentrations in O_2_ stream at 0.2, 0.5 and 1.0 A·cm^−2^ and comparison to those in the literature [15,16].

		j (A·cm^–2^)	H_2_ Volume Fraction in O_2_ / %		
			Experimental	Literature	
60 °C	BoT	0.2	1.1 ± 0.002	~1.5 %	Omrani et al. (2021)
		0.5	0.8 ± 0.001	~1.1 %	
		1.0	0.4 ± 0.004	~0.9 %	
	EoT	0.2	0.9 ± 0.003	-	
		0.5	0.8 ± 0.001	-	
		1.0	0.4 ± 0.003	-	
80 °C	BoT	0.2	0.8 ± 0.005	~0.9 %	Martin et al. (2022)
		0.5	0.5 ± 0.004	~0.6 %	
		1.0	0.4 ± 0.003	~0.5 %	
	EoT	0.2	0.9 ± 0.006	-	
		0.5	0.7 ± 0.006	-	
		1.0	0.5 ± 0.002	-	

### 3.3. Fluoride Emission Rate

The calibration series for the photometric detection of the FER was adapted to a random sample survey during the measurements and consisted of the following concentrations: 0 mg·L^−1^, 0.02 mg·L^−1^, 0.05 mg·L^−1^, 0.2 mg·L^−1^ and 0.5 mg·L^−1^. The area-specific FER was calculated from the amount of fluoride released for the active CCM according to Equation (3).

FER = c_F_/M_F_ × V_water_/A_cell_/n_sampling_ (3)

**Figure 7:**
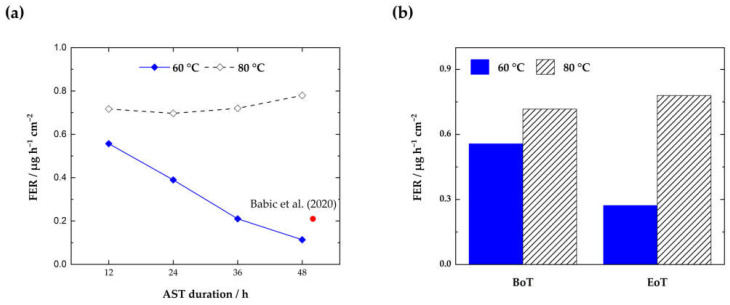
Area-specific fluoride emission rate (FER) during the AST at 60 °C and 80 °C (**a**) and cumulative F^−^ concentrations (**b**) from the anode compartment at the beginning and at the end of testing (BoT/EoT). Red marker indicates a FER value from the literature (Nafion™ 115 at 60 °C) [40].

For the calculation of the FER (${µg}_{F^{-}}$h^−1^·cm^−2^); the fluoride concentration in the water sample, c_F_ (mg·L^−1^); the molar mass of fluoride, M_F_ (g·mol^−1^); the volume of the exhaust water, V_water_ (L); the active cell area, A_cell_, (cm^2^); and the sampling time, n_sampling_ (hours) need to be considered [27]. In Figure 7, the FER during the ASTs from the two cells tested at 60 °C and 80 °C and the cumulative FER at BoT and EoT are displayed.

The measurements show a constantly higher FER for the cell tested at 80 °C. Here, the highest F^−^ concentration is observed after 50 h, which indicates that even more detrimental membrane ageing is possible with longer operation in AST mode. An opposing trend is visible for the cell tested at 60 °C. Whereas the cumulative FER values at 80 °C (see Figure 7b) align with approximately 3 ${µg}_{F^{-}}\;$h^−1^·cm^−2^, a significant decrease in the FER can be observed at 60 °C. At the lower temperature, the maximum FER is observed in the first 12 h (0.56 ${µg}_{F^{-}}$ h^−1^·cm^−2^). In the following hours, the F^−^ concentration from the anode H_2_O decreases by 52% to 0.27 ${µg}_{F^{-}}\;$h^−1^·cm^−2^ after 50 h.

Fouda-Onana et al. [21] reported a similar behavior in ageing protocols performed at 60 °C and 80 °C. In their test conditions, temperature was found to be more prejudicial for membrane degradation compared to the operating current. The overall higher FER at 80 °C can be seen as an indicator for membrane degradation, which is consistent with other outcomes presented in this paper.

### 3.4. Correlation of Fluoride Emission and Hydrogen Crossover

Chemical degradation in PEMWE is based on the reaction of in-situ generated radicals with the PFSA membrane. The mechanism for the creation of radicals and subsequent degradation is described in reactions I–V in the introduction. In idle mode (OCV), gas crossover becomes predominant since the active transport of H^+^ through the membrane is not actively promoted as part of the electrochemical reaction anymore. On the anode of the electrolyzer, a chemical recombination of hydrogen and oxygen from H_2_ crossover can take place according to reactions VI–VIII.

H_2_ → 2H• (via Pt catalyst) (VI)

H• (diffused through PEM to anode) + O_2_ → HO_2_• (VII)

HO_2_• + H• → H_2_O_2_ (diffuses into PEM) (VIII)

**Figure 8:**
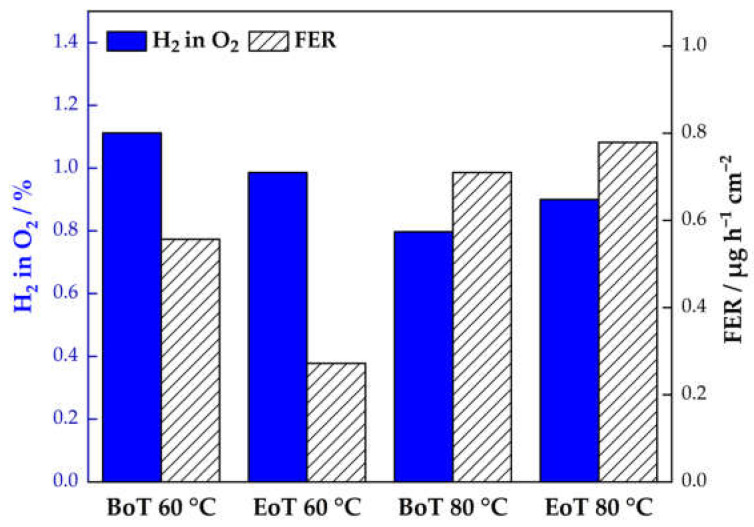
Fluoride emission rate (FER) and H_2_ concentration in O_2_ at 0.2 A·cm^−2^ at begin of testing (BoT) and end of testing (EoT).

For this recombination, catalyst sites need to be present to facilitate the reaction at significant rates. Hydrogen is split into hydrogen radicals on the catalyst surface (VI), which diffuse through the PEM, where they react with oxygen (VII). The formed hydroperoxide radical (HO_2_•) can either directly attack the membrane or further react with a hydrogen radical to H_2_O_2_ (VIII). In this study, we have identified a correlating trend between the FER and the H_2_ crossover over the course of the ASTs at 60 °C and 80 °C (Figure 8).

For reactions on the anode that are based on H_2_ crossover, the limiting step is always the gas diffusion process. Higher diffusion rates and a higher gas crossover will therefore lead directly to higher radical generation and chemical degradation. Therefore, it can be concluded that there is a direct correlation between the H_2_ in O_2_ content and the FER in the effluent anode water of a PEMWE. This assumption is supported by the findings of this study. As presented in Figure 8, the FER at 60 °C shows the highest value in the beginning and drops by about 52% at EoT. This behavior is consistent with the results from the crossover measurements. Here, the H_2_ in O_2_ content is higher compared to EoT (22% decrease at 0.2 A·cm^−2^). At 80 °C, the gas crossover and the FER again act consistently. The gas crossover increases by ~13%, and the cumulative FER increases by 8.5% over the course of the experiment. The findings therefore show a direct correlation between crossover H_2_ and the formation of detrimental radicals that accelerate chemical membrane degradation. This implies that gas purity can be used as an indicator for ongoing membrane degradation and vice versa. Furthermore, a direct effect of temperature and chemical membrane ageing has been reported.

### 3.5. Scanning Electron Microscopy

**Figure 9:**
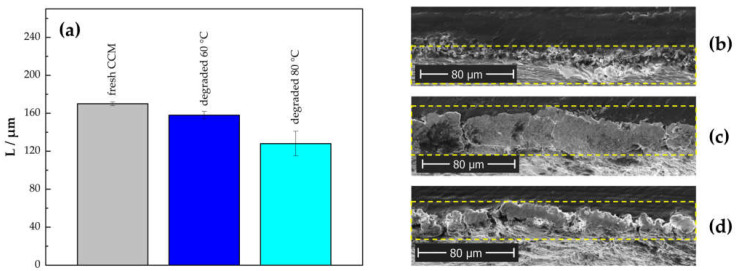
Comparison of the averaged membrane thicknesses (**a**) and cross-sections of the Ir catalyst layers of a fresh CCM (**b**) after the AST at 60 °C (**c**) and after the AST at 80 °C (**d**).

**Figure 10:**
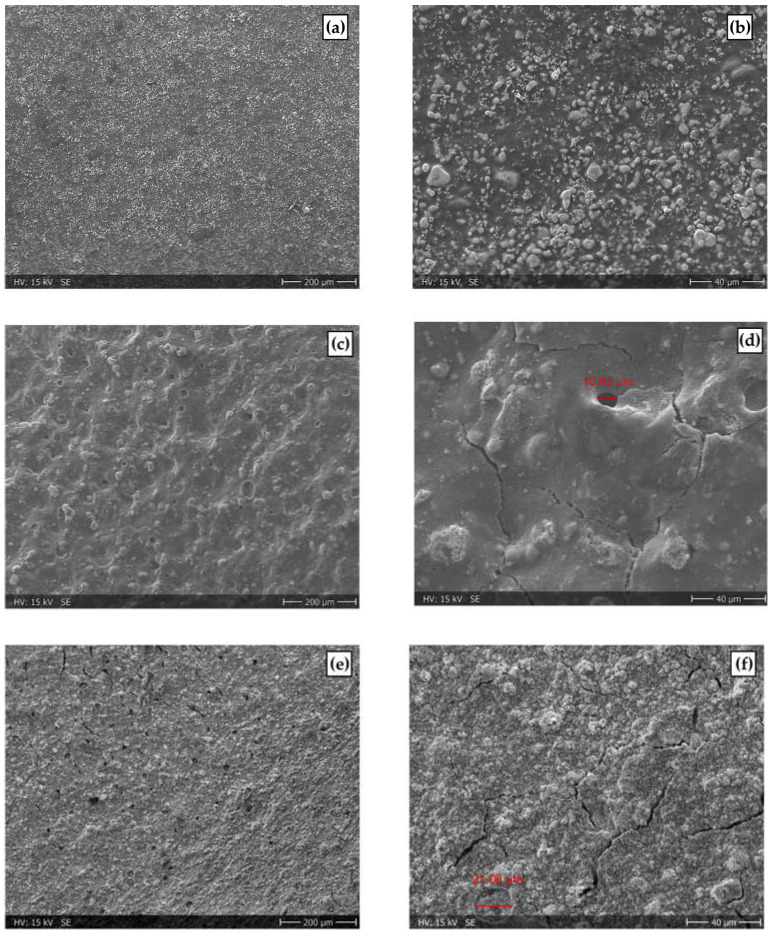
Surface morphologies of the Ir anode from a fresh sample with ×100 magnification (**a**) and ×500 magnification (**b**) and degraded CCMs at 60 °C ×100 magnification (**c**) and ×500 magnification, red marker indicating the size of formed hole (**d**) and degraded at 80 °C ×100 magnification (**e**) and ×500 magnification, red marker indicating size of formed crater (**f**).

In order to analyze the membrane thinning effect after the ASTs, scanning electron microscopy (SEM) cross-sectional images of two samples post-operation (AST at 60 °C and AST at 80 °C) and one fresh MEA were recorded (Figure S1 in the Supplementary). A comparison of the averaged membrane thicknesses and cross-sections of the Ir-catalyst layers are presented in Figure 9. Additionally, to analyze the morphological changes after testing, surface images of the anode CL were recorded (see Figure 10).

#### 3.5.1. Cross-sectional Analysis

The average of the membrane thicknesses presented Figure 9a shows a significantly higher degree of thinning for the sample tested at 80 °C (~25% at 80 °C compared to ~7% at 60 °C). This correlates with the results from the FER that indicate a higher amount of F^−^ release during the AST conducted at 80 °C. 

Compared to a fresh CCM in Figure 9b, the CL cross-section image of the CCM from the AST conducted at 60 °C (Figure 9c) indicates that a mixed layer of catalyst, ionomer, and membrane has formed. The reduced interfacial resistance of this more homogeneous phase might explain the reduced HFR and improved electrochemical behavior measured in the polarization curves. An opposite effect for the CCM tested at 80 °C might be responsible for the measured performance decay in this cell. 

Membrane thinning as a result of a radical attack is widely known and has been reported by several researchers before [10,19,21,41,42,43,44]. According to Kusoglu et Weber [10], the chemical and mechanical degradation of PFSA membranes is inextricably linked and dependent on operation and dominant stressors. The induced crossover conditions in the presented ASTs could therefore magnify the degree of membrane thinning in a short period of time.

#### 3.5.2. Surface Morphology Analysis

Figure 10a–f shows the surface morphologies of the analyzed anode CLs before and after the ASTs with 500x and 100x magnification.

Images of the anode CL surface presented in Figure 10c–f reveal a significant change in morphology for both ASTs. Pinholes of approximately 10–20 µm in size and cracks in the CL are visible and show that overall cell ageing occurred during the ASTs. In Figure 10c,d, the CL of the cell tested at 60 °C is shown. Compared to the fresh CCM, the surface appears to be much smoother. Figure 10e,f shows the SEM images obtained from the CCM tested at 80 °C. Herein, the CL surface shows a markedly different morphology compared to the CCM tested at 60 °C. The surface has a rougher appearance, and the cross-sectional image (Figure 9d) reveals that some parts of the CL are already delaminated. The surface morphology analysis aligns with the results from the cross-sectional images of the CCMs. For the cell tested at 60 °C a mixed layer of catalyst, ionomer and membrane was identified that is characterized by a smooth surface appearance. For the cell tested at 80 °C, the surface appears much rougher, and the delaminated parts of the CL identified in the cross-sectional images might be responsible for the overall worse performance of this cell. The optical investigations support the findings presented in the previous sections (3.1–3.3) of this work and provide valuable insights into membrane ageing processes triggered by H_2_ crossover in PEMWE.

## 4. Conclusions

An AST for the chemical degradation of the membrane in PEMWE was developed, which triggers the H_2_ crossover in idle mode and accelerates the ageing of the PFSA membranes. The AST profile comprises of a 50 h hold period at OCV and was conducted at two different temperature setpoints (60 °C and 80 °C, respectively).

An increase in the HFR was observed for both temperatures, whereas the cumulative fluoride emission rate after the AST was 2.8-fold higher for CCMs subjected to the AST at 80 °C. This observation is clearly linked to the increased chemical degradation of the membrane at higher temperatures. The H_2_ crossover determined by gas chromatography confirms the findings. The H_2_ content in the O_2_ stream increased at 80 °C, whereas a more constant behavior and even decrease in the H_2_ crossover at low current densities was observed at 60 °C. The analysis of the morphology of the CCMs via SEM revealed detrimental changes in the anode catalyst layer connected to the membrane’s chemical degradation. Furthermore, a significant 25% degree of thinning was observed for the cell tested at 80 °C, compared to 7% at 60 °C. H_2_ crossover was identified as a key factor for membrane degradation. The new AST protocol is a promising method to compare membrane degradation from a single cell to the system.

## Figures and Tables

**Table 1:** Comparison of the EIS data recorded at 1.0 A·cm^−2^ and the slope of the i/E curve in the ohmic region of the polarization curve (between 0.6 and 1.4 A·cm^−2^).

		Slope i/E Curve (Ω·cm^2^)	High-Frequency Resistance (HFR) (Ω·cm^2^)	Low-Frequency Resistance (LFR) (Ω·cm^2^)
60 °C	BoT	0.15	0.17	0.26
	EoT	0.16	0.17	0.27
80 °C	BoT	0.15	0.15	0.23
	EoT	0.16	0.16	0.24

## Data Availability

The data that support the findings of this study are available within the article.

## Supplementary Materials

The following supporting information can be downloaded at: https://www.mdpi.com/article/10.3390/membranes13030348/s1, Figure S1: Cross-sectional images of the CCMs.
