# Supplementary figures and images for: Analysis of PEM Water Electrolyzer Failure Due to Induced Hydrogen Crossover in Catalyst-Coated PFSA Membranes
Source: Membranes (Basel). 2023 Mar 17;13(3):348. doi: 10.3390/membranes13030348 (PMC10053853; doi:10.3390/membranes13030348)

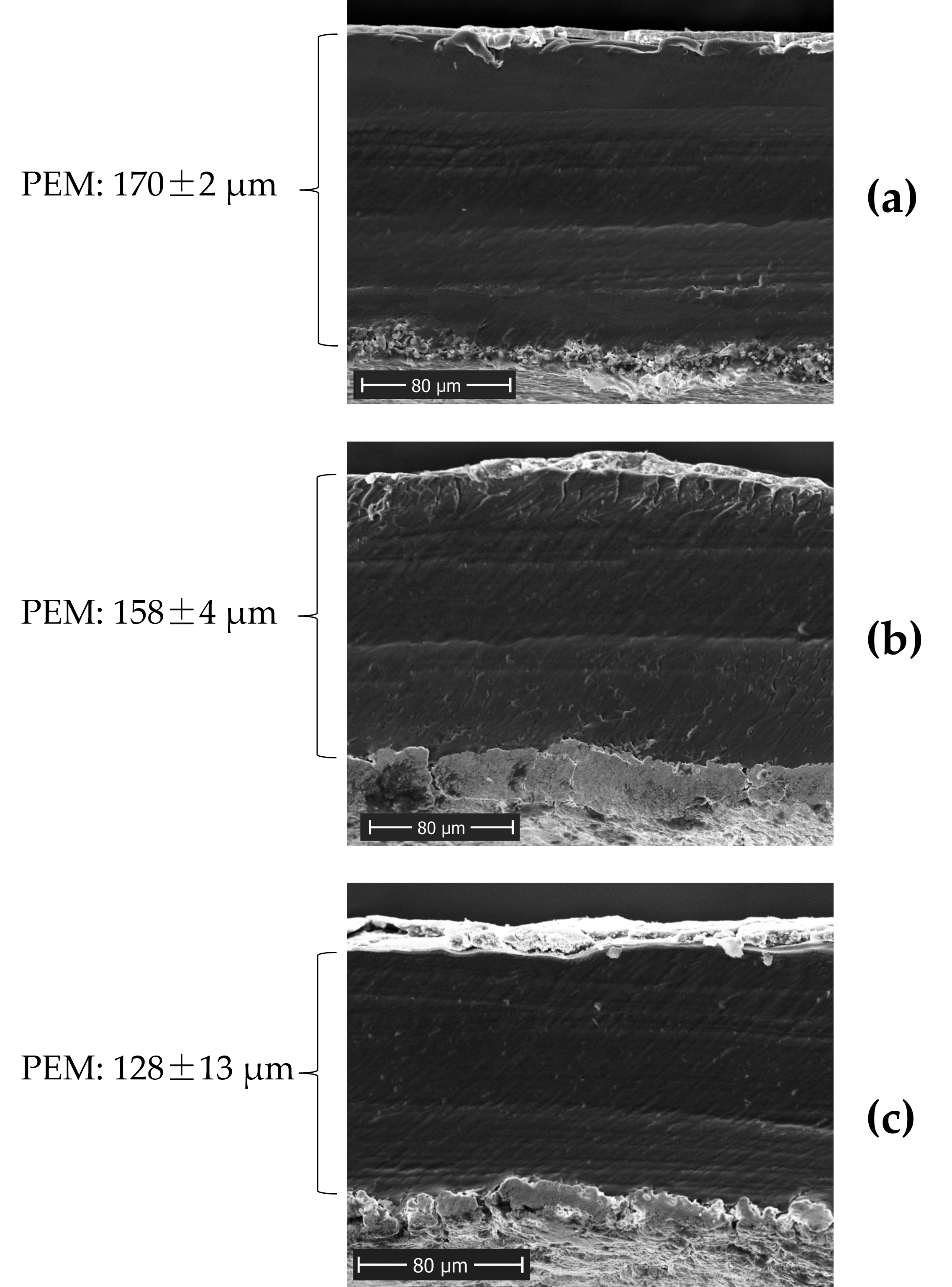

Supplement: Supplementary file 1 [file membranes-13-00348-s001.zip › membranes-2282534-supplementary.png]
